# Peptide-Reactive T-cell Response as a Novel Biomarker in Patients with Head and Neck Cancer Treated with Anti–PD-1 Antibody

**DOI:** 10.1158/2767-9764.CRC-25-0796

**Published:** 2026-07-13

**Authors:** Takumi Kumai, Shota Sakaue, Hisataka Ominato, Takahiro Inoue, Ryosuke Sato, Risa Wakisaka, Hiroki Komatsuda, Ryusuke Hayashi, Michihisa Kono, Hidekiyo Yamaki, Kenzo Ohara, Kan Kishibe, Miki Takahara

**Affiliations:** 1 Department of Innovative Head and Neck Cancer Research and Treatment (IHNCRT), Asahikawa Medical University, Asahikawa, Japan.; 2 Department of Otolaryngology, Head and Neck Surgery, Asahikawa Medical University, Asahikawa, Japan.

## Abstract

Immune checkpoint inhibitors have improved outcomes in recurrent or metastatic head and neck squamous cell carcinoma (R/M HNSCC), yet reliable biomarkers predicting response to programmed death 1 (PD-1) blockade remain limited. We retrospectively analyzed 40 patients with R/M HNSCC treated with nivolumab or pembrolizumab monotherapy, evaluating clinical data, laboratory parameters, PD-L1 expression, tumor-infiltrating T cells, and peripheral immune features. Peripheral blood mononuclear cells obtained before treatment were assessed by flow cytometry for exhaustion markers and short-term tumor antigen–derived peptide stimulation with IFN-γ ELISA. The objective response rate was 18%, with median overall survival of 13 months and progression-free survival of 3 months. PD-L1 expression and densities of CD4^+^, CD8^+^, and FoxP3^+^ T cells were not associated with response. In contrast, responders more frequently developed immune-related adverse events and had preserved cervical lymph nodes. Favorable responses were associated with higher baseline lymphocyte and lower neutrophil percentages, as well as lower frequencies of CD38^+^ CD8^+^ T cells. Notably, c-Met–derived peptide stimulation induced significantly higher IFN-γ production in responders, indicating the presence of circulating tumor antigen–reactive T cells. These findings suggest that tumor antigen–reactive T cells, together with a favorable systemic immune profile, are associated with clinical benefit from PD-1 blockade and that peripheral blood–based peptide-reactive T-cell assays may provide a practical approach for biomarker development in R/M HNSCC.

**Significance:**

c-Met–specific circulating tumor antigen–reactive T cells predict response to PD-1 blockade in R/M HNSCC. Favorable systemic immunity further supports outcomes. Peripheral blood–based assays offer a practical, noninvasive biomarker to identify patients most likely to benefit from immunotherapy.

## Introduction

Immunotherapy represents a unique therapeutic modality for cancer in addition to surgery, radiotherapy, and chemotherapy. The clinical implementation of immune checkpoint inhibitors (ICI) has markedly transformed the management of recurrent or metastatic head and neck squamous cell carcinoma (R/M HNSCC). Human papillomavirus (HPV)-positive HNSCC is generally regarded as an immune-hot tumor, and similarly, HPV-negative HNSCC frequently harbors p53 mutations that give rise to abundant neoantigens, rendering it immunologically active as well. Accordingly, ICIs have demonstrated promising efficacy across HNSCC, including in HPV-negative disease (1–3).

In current clinical practice, the principal targets of ICIs are programmed death 1 (PD-1) and its ligand PD-L1, which function as negative immune checkpoints. Although PD-L1 expression on tumor cells and infiltrating immune cells has been considered a potential predictor of response to PD-1 blockade (2), neither PD-L1 expression nor HPV status reliably correlates with clinical efficacy (4). Moreover, we previously reported that PD-L1 expression frequently differs between primary and R/M lesions (5), indicating that PD-L1 assessment using primary tumor samples may not accurately reflect the immunologic landscape in R/M HNSCC. Because biopsy acquisition from R/M sites is often challenging, there is an urgent need for biomarkers that can predict the real-time status of antitumor immunity.

In this study, we aimed to identify prognostic pretreatment biomarkers for ICI therapy in patients with R/M HNSCC by analyzing peripheral blood and clinical specimens. Building on our previous work identifying tumor antigen–derived epitopes and circulating precursors of antigen-reactive T cells in HNSCC (6–8), we investigated whether the presence of tumor antigen–reactive T cells in the peripheral blood serves as a prognostic indicator for clinical outcomes following PD-1 blockade.

## Materials and Methods

### Patients

A total of 40 patients with R/M HNSCC who received nivolumab or pembrolizumab monotherapy at Asahikawa Medical University were included. All enrolled participants finished the study, i.e., there was no attrition. Patients treated with pembrolizumab in combination with chemotherapy were excluded. Prior radiation was defined as definitive radiotherapy to the head and neck region. No patients received palliative radiotherapy. Best overall response was assessed using the Response Evaluation Criteria in Solid Tumors and categorized as complete response (CR), partial response (PR), stable disease (SD), or progressive disease (PD). CR and PR were classified as responders, whereas SD and PD were classified as nonresponders. Overall survival (OS) and progression-free survival (PFS) were calculated from the initiation of PD-1 blockade to death or disease recurrence. Radiologic evaluation using CT was performed approximately 2 months after treatment initiation and subsequently at 3-month intervals. Clinical follow-up, including local examinations, was conducted monthly during the first year and every 2 to 3 months thereafter.

Laboratory tests—including albumin, cholinesterase, C-reactive protein (CRP), hemoglobin, neutrophil-to-lymphocyte ratio (NLR), platelet count, SCC antigen, total protein, and white blood cell count—were performed 1 week before treatment, 1 week after treatment, and 1 month after treatment.

### Immunohistochemical staining

Immunohistochemical staining of clinical formalin-fixed, paraffin-embedded specimens was performed using the EnVision HRP System (Dako). Sections were boiled in citrate buffer (pH 6.0) for antigen retrieval and incubated with 1% H_2_O_2_ in methanol to block endogenous peroxidase activity. After incubation with the following primary antibodies—rabbit anti-human c-Met polyclonal antibody (C28, RRID: AB_631941), mouse anti-human CD4 (MT310, RRID: AB_627055), mouse anti-human Foxp3 (2A11G9, RRID: AB_783444), mouse anti-human CD8 (MCD8, RRID: AB_629215), mouse anti-human CD56 (123A8, RRID: AB_784749), mouse anti-human PD-1 (J116), and mouse anti-human PD-L1 (D-8)—and applied for 1 hour at room temperature, sections were incubated with HRP-conjugated secondary antibodies and substrate. All primary antibodies except anti-human PD-1 were purchased from Santa Cruz Biotechnology; anti-human PD-1 was purchased from Bio X Cell.

The quantity score was based on the percentage of positively stained cells and categorized as 0 (<5%), 1 (5%–25%), 2 (26%–50%), or 3 (51%–100%).

### Peptide stimulation of peripheral blood mononuclear cells

Peripheral bloodmononuclear cells (PBMC) collected 1 week before treatment using Lymphoprep (Serumwerk Bernburg) were divided into two equal portions for functional assays. One portion was stimulated with or without EGFR_875–889_ or c-Met_817–831_ peptides (Hokkaido System Science), which are known to induce HNSCC-reactive T cells (6, 7), and cultured for 7 days. PADRE (aK‐Cha‐VAAWTLKAAa, in which “a” denotes D‐alanine and “Cha” denotes L‐cyclohexylalanine) peptide that can bind with multiple HLA-DR molecules was used as a positive control (9). The remaining portion was cryopreserved and later γ-irradiated to serve as antigen-presenting cells, which were added on day 8. Human IL-2 (10 IU/mL; Peprotech) was added on day 10. Supernatants were collected and assessed for IFN-γ production using ELISA (BD PharMingen).

### Flowcytometry

Antibodies for flow cytometry were purchased from BioLegend. PBMCs collected 1 week before treatment were stained with anti-CD4–PE (OKT4, AB_571955), anti-CD8–FITC (HIT8a, RRID: AB_756152), anti-PD-1–APC (EH12.2H7, RRID: AB_940475), anti-ICOS–APC (C398.4A, RRID: AB_416334), anti-Tim-3–APC (F38.2E2, RRID: AB_2561718), anti-LAG-3–APC (7H2C65, RRID: AB_2728373), anti-CD56–PE (MEM188, RRID: AB_314448), or anti-CD38–APC (HIT2, RRID: AB_2904466). Stained cells were analyzed using a CytoFLEX flow cytometer (Beckman Coulter). The positivity of each molecule was defined using thresholds established based on negative control samples (unstained or isotype controls), with gates set to exclude background fluorescence. Cells exceeding this threshold were considered positive. Sequential gating was performed on lymphocytes (Forward Scatter/Side Scatter), singlets, and CD4^+^/CD8^+^ T cells prior to analysis (Supplementary Fig. S1).

### Ethical consideration

Approval for data collection and analysis was obtained from the Asahikawa Medical University Institutional Review Board (#16217). All procedures involving human participants followed the ethical standards of the institutional and/or national research committee and the principles of the 1964 Declaration of Helsinki and its later amendments. Written informed consent was obtained from all participants.

### Statistical analysis

Survival and recurrence curves were estimated using the Kaplan–Meier method, and differences were evaluated using the log-rank test. Other variables were compared using Fisher exact test or the Mann–Whitney U test. Statistical significance was defined as *P* < 0.05.

## Results

### Survival outcomes and treatment response in patients receiving anti–PD-1 antibody

**Figure 1.**
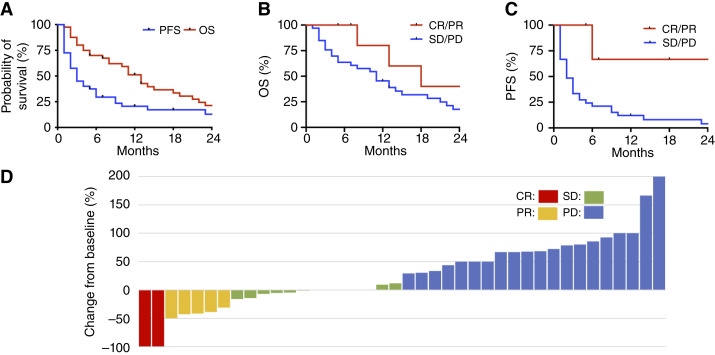
Survival outcomes and treatment response in patients receiving anti–PD-1 antibody. **A,** Kaplan–Meier curves for PFS and OS in the entire cohort. **B,** OS stratified by treatment response, showing better improved survival in patients achieving CR/PR compared with those with SD/PD. **C,** PFS stratified by treatment response, demonstrating significantly prolonged PFS in patients with CR/PR relative to SD/PD. **D,** Waterfall plot illustrating the maximum percentage change in tumor size from baseline for each patient. Bars are color-coded according to best overall response (red, CR; yellow, PR; green, SD; and blue, PD).

In this study, we analyzed 40 patients with R/M HNSCC who received ICI monotherapy. Patients treated with ICI in combination with cytotoxic chemotherapy were excluded to ensure that the therapeutic efficacy of ICI monotherapy could be evaluated without confounding effects. Their age varied from 39 to 84 years (mean age, 71 years). All patients received anti–PD-1 therapy in the second-line or later setting. Of these, 29 patients (72%) were treated in the second-line setting, whereas 11 patients (28%) received treatment in the third-line or later setting. As summarized in Table 1, the objective response rate was 18%, consistent with previously reported phase III trials of nivolumab and pembrolizumab (2, 3). The median OS and PFS were 13 and 3 months, respectively (Fig. 1A). Median OS tended to be prolonged in responders (CR/PR) compared with nonresponders (SD/PD) at 18 months versus 11 months (Fig. 1B). The corresponding 1-year OS rates were 71% and 45%, respectively. PFS was significantly longer among responders than nonresponders (*P* = 0.003; Fig. 1C). Among the 40 patients, 31 received nivolumab and 9 received pembrolizumab. Clinical responses were observed in five patients treated with nivolumab (15%) and two patients treated with pembrolizumab (22%). CR was achieved in two patients (5%; Fig. 1D).

**Table 1.** Clinical characteristic of patients.

Parameters	Total	CR/PR (*n* = 7)	SD/PD (*n* = 33)	*P* value
Age (median)	39–84 (71)	39–80 (76)	45–84 (69)	0.89
Male:female	6:34	2:5	4:29	0.18
Local recurrence	30/40 (75%)	4/7 (57%)	26/33 (79%)	0.23
Nodal recurrence	4/40 (10%)	0/7 (0%)	4/33 (12%)	0.33
Distant metastasis	13/40 (33%)	3/7 (43%)	10/33 (30%)	0.52
History of neck dissection	9/40 (23%)	0/7 (0%)	9/33 (27%)	0.12
History of radiation	35/40 (88%)	7/7 (100%)	28/33 (85%)	0.27
History of cetuximab	8/40 (20%)	1/7 (14%)	7/33 (21%)	0.67
Third or later line	11/40 (28%)	2/7 (29%)	9/33 (27%)	0.94
Number of course	1–80 (6)	5–80 (10)	1–32 (5)	**0.04**
≥Grade 3 irAE	9/40 (23%)	3/7 (43%)	6/33 (18%)	0.16
TPS	0–80 (5.5)	1–80 (10)	0–80 (15)	0.86
CPS	0–80 (15)	1–80 (10)	0–80 (15)	0.98

Bold values indicate statistical significance (P < 0.05).

We further evaluated clinical parameters associated with therapeutic response. As shown in Table 1, age and sex distributions were comparable between responders and nonresponders. Patterns of recurrence or metastasis were not associated with objective response to anti–PD-1 therapy. Prior exposure to radiation or systemic chemotherapy, including cetuximab, did not seem to influence treatment response. Notably, none of the responders had a history of neck dissection. Immune-related adverse events (irAE) occurred more frequently in responders (43%) than in nonresponders (18%); however, this difference did not reach statistical significance. Only grade ≥3 irAEs were included in the analysis, and all observed irAEs were grade 3. In the responder group, irAEs included colitis, interstitial pneumonitis, and liver dysfunction, whereas in the nonresponder group, irAEs included dermatitis, interstitial pneumonitis, cholangitis, hypothyroidism, and fulminant type 1 diabetes mellitus.

### Immunohistochemical staining for T-cell subsets in responders and nonresponders to anti–PD-1 therapy

Because PD-1 interacts with PD-L1, the expression of PD-L1 on tumor cells alone (tumor proportion score; TPS) or on both tumor and immune cells (combined positive score; CPS) has been considered a potential surrogate marker for predicting clinical responses to anti–PD-1 antibodies. However, in our cohort, neither TPS nor CPS was associated with treatment response (Table 1). To further characterize the tumor immune microenvironment, we performed immunohistochemical analyses of T-cell subsets and immune-related molecules.

**Figure 2.**
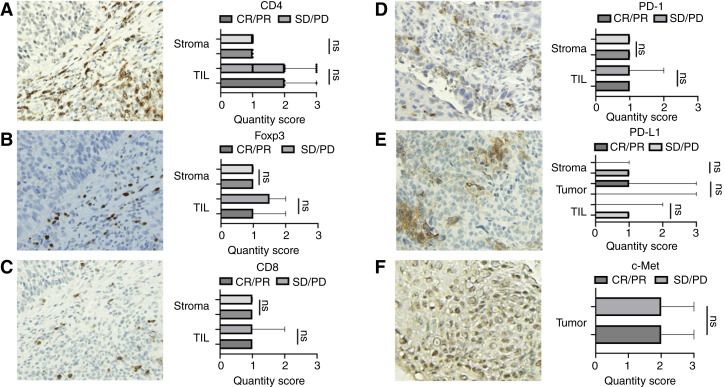
Immunohistochemical staining for T-cell subsets in responders (CR/PR) and nonresponders (SD/PD). Representative immunohistochemical images of (**A**) CD3, (**B**) CD4, (**C**) CD8, (**D**) PD-1, (**E**) PD-L1, and (**F**) c-Met staining in tumor tissues from responders (CR/PR) and nonresponders (SD/PD). Cases with no detectable staining are shown as zero values, which may appear as missing bars. Magnification: ×200. ns, not significant.

CD4^+^ T cells, including FoxP3^+^ regulatory T cells, were identified within both stromal regions and tumor-infiltrating lymphocytes (TIL), but their densities showed no correlation with response status (Fig. 2A and B). Similarly, the abundance of CD8^+^ effector T cells was comparable between responders and nonresponders in both the stromal compartment and TILs (Fig. 2C). NK cells were rarely observed in either region. Furthermore, the expression of PD-1 and PD-L1—key molecules targeted by ICIs—did not differ between the two groups (Fig. 2D and E). Expression levels of tumor-associated antigens, including c-Met (Fig. 2F) and EGFR (Supplementary Fig. S2), were also similar regardless of treatment response.

### Laboratory parameters between anti–PD-1 antibody responders (CR/PR) and nonresponders

**Figure 3.**
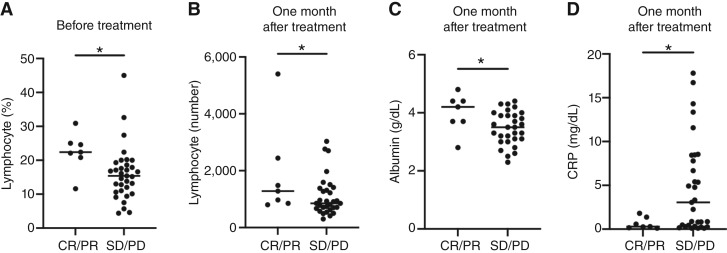
Comparison of laboratory parameters between responders (CR/PR) and nonresponders (SD/PD). **A,** Pretreatment lymphocyte percentage was significantly higher in the CR/PR group than in the SD/PD group. **B,** Lymphocyte count 1 month after treatment was also significantly higher in CR/PR compared with SD/PD. **C,** Serum albumin level 1 month after treatment showed a significantly higher value in CR/PR than in SD/PD. **D,** CRP level 1 month after treatment was significantly lower in CR/PR than in SD/PD. Horizontal bars indicate median values. *, *P* < 0.05.

To evaluate systemic factors that may influence the response to anti–PD-1 therapy, we compared blood-based laboratory parameters between responders and nonresponders at three time points: 1 week before treatment, 1 week after treatment, and 1 month after treatment. As shown in Table 2 and Fig. 3A, a higher lymphocyte and a lower neutrophil percentage prior to treatment were significantly associated with a favorable response to anti–PD-1 therapy. Other baseline parameters, including NLR, platelet count, modified Glasgow prognostic score, prognostic nutritional index, and albumin level, were not associated with treatment response. Likewise, none of the parameters measured 1 week after treatment correlated with clinical outcomes. At 1 month after treatment, higher absolute lymphocyte counts and higher albumin levels, as well as lower CRP levels and higher prognostic nutritional index, were significantly associated with favorable responses (Fig. 3B–D).

**Table 2.** Laboratory data of patients.

One week before treatment				One week after treatment				One month after treatment			
Parameters	CR/PR (*n* = 7)	SD/PD (*n* = 33)	*P* value	Parameters	CR/PR (*n* = 7)	SD/PD (*n* = 33)	*P* value	Parameters	CR/PR (*n* = 7)	SD/PD (*n* = 33)	*P* value
WBC	3,210–6,380 (5,550)	1,290–18,160 (5,590)	0.24	WBC	3,600–7,380 (6,090)	1,660–16,180 (5,615)	0.53	WBC	4,380–7,830 (5,730)	1,710–16,810 (7,050)	0.17
Lymphocyte	430–1,880 (1,230)	340–3,510 (880)	0.75	Lymphocyte	500–1,770 (880)	380–2,120 (820)	0.76	Lymphocyte	680–5,400 (1,280)	300–3,030 (850)	**0.04**
Neutrophil	2,110–4,860 (3,300)	570–15,960 (3,970)	0.16	Neutrophil	2,750–5,130 (4,300)	840–13,690 (4,030)	0.46	Neutrophil	2,260–5,790 (4,250)	1,010–15,550 (5,170)	0.12
Eosinophil	40–410 (50)	0–860 (120)	0.88	Eosinophil	40–400 (80)	0–500 (120)	0.98	Eosinophil	60–790 (120)	0–1,250 (160)	0.78
Basophil	20–70 (30)	0–200 (20)	0.91	Basophil	20–50 (30)	0–100 (30)	0.79	Basophil	30–70 (40)	10–110 (30)	0.71
Lymphocyte %	11.6–30.9 (22.4)	4.4–45 (15.4)	**0.04**	Lymphocyte %	9.7–31.3 (16.4)	5.5–37.3 (16.6)	0.88	Lymphocyte %	9.6–33.8 (18.9)	3–33.8 (14.9)	0.18
Neutrophil %	59.4–76.2 (63.8)	44.1–88.6 (73.5)	**0.03**	Neutrophil %	58.7–82.4 (68)	50.7–88.5 (70)	0.77	Neutrophil %	51.6–79.4 (64.3)	56.2–92.5 (73.8)	0.08
Eosinophil %	0.8–6.9 (1.3)	0–18.9 (2)	0.76	Eosinophil %	0.5–7.4 (1.4)	0–9.7 (2.7)	0.89	Eosinophil %	0.8–10.1 (2.4)	0–14.1 (2.1)	0.52
Basophil %	0.4–6 (0.5)	0–2 (0.4)	0.06	Basophil %	0.3–0.8 (0.6)	0–1.9 (0.5)	0.72	Basophil %	0.4–0.9 (0.7)	0.1–1.3 (0.4)	0.17
NLR	1.9–6.6 (2.8)	0.2–20 (4.7)	0.16	NLR	1.9–8 (4.2)	1.4–16 (4.2)	0.53	NLR	0.6–7.7 (2.9)	1.8–31.1 (4.9)	0.12
Hb	10.2–13.8 (11.6)	8–15.4 (11.5)	0.61	Hb	10.3–12.3 (11.8)	7.9–15.4 (10.6)	0.47	Hb	9.9–15.8 (11.7)	7.8–13.8 (10.7)	0.16
Plt (×10E4)	16.4–42.2 (22.2)	14.7–47.3 (22.2)	0.73	Plt (×10E4)	14.1–37.3 (22.5)	9.5–43 (23.9)	0.37	Plt (×10E4)	14.4–38.9 (21.5)	7.9–54.2 (25.4)	0.44
TP	5.5–7.6 (7.3)	4.8–7.6 (6.6)	0.37	TP	5.9–7.3 (6.7)	5.4–7.5 (6.7)	0.49	TP	6–7.4 (7.2)	5.8–7.8 (6.8)	0.80
Alb	2.8–4.8 (4.2)	2.1–4.8 (3.7)	0.18	Alb	2.9–4.5 (3.7)	2.4–4.2 (3.5)	0.26	Alb	2.8–4.8 (4.2)	2.3–4.4 (3.5)	**0.04**
ChE	127–352 (267)	81–416 (228)	0.75	ChE	143–331 (226)	84–458 (223)	0.78	ChE	123–341 (260)	102–415 (210)	0.38
CRP	0.1–2.8 (0.46)	0.1–12 (1)	0.26	CRP	0.2–6.9 (1.3)	0.1–17.7 (1.4)	0.47	CRP	0.1–1.92 (0.29)	0.1–17.8 (3.06)	**0.04**
Modified GPS	0–2 (0)	0–2 (1)	0.72	Modified GPS	0–2 (1)	0–2 (1)	0.95	Modified GPS	0–1 (0)	0–2 (1)	0.06
PNI	32–55 (49)	26–56 (42)	0.2	PNI	32–53 (44)	28–47 (41)	0.3	PNI	32–69 (51)	27–52 (40)	**0.007**
CRP/Alb	0.02–1 (0.11)	0.03–4 (0.29)	0.29	CRP/Alb	0.05–2.38 (0.41)	0.02–3.14 (0.29)	0.59	CRP/Alb	0.02–0.48 (0.07)	0.02–7.12 (1.07)	0.05
SCC	1.7–7.1 (3.1)	0.9–20.1 (2.1)	0.88	​	​	​	​	SCC	0.9–3.4 (2.1)	1.2–79.9 (4.3)	0.28

Abbreviations: Alb, albumin; ChE, choline esterase; GPS, Glasgow prognostic score; Hb, hemoglobin; Plt, platelet; PNI, prognostic nutritional index; TP, total protein; WBC, white blood cell. Bold values indicate statistical significance (P < 0.05).

### Comparison of peripheral lymphocyte markers between responders and nonresponders to anti–PD-1 therapy

**Figure 4.**
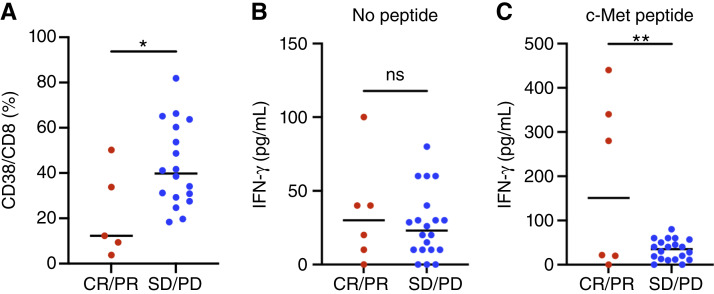
IFN-γ production by PBMCs from responders and nonresponders with or without c-Met peptide stimulation. **A,** Scatter plots showing the expression of CD38 in CD8^+^ T cells evaluated by flow cytometry in responders (red, CR/PR) and nonresponders (blue, SD/PD). Horizontal bars represent median values. Statistical differences were assessed using the Mann–Whitney U test. *, *P* < 0.05. **B,** PBMCs isolated from patients were cultured without peptide stimulation, and IFN-γ concentrations in the supernatant were measured by ELISA. IFN-γ levels did not differ significantly between the CR/PR and SD/PD groups. ns: not significant. **C,** PBMCs were stimulated with the c-Met–derived peptide, resulting in a significantly higher IFN-γ production in the CR/PR group compared with the SD/PD group (*P* < 0.01). Each dot represents an individual patient sample, and horizontal bars indicate median values. ns, not significant. **, *P* < 0.01.

To assess the exhaustion status of circulating lymphocytes, we analyzed the expression of inhibitory immune checkpoints on peripheral blood lymphocytes by flow cytometry. In responders, PD-1 expression on both CD4^+^ and CD8^+^ T cells was detectable but comparable with that observed in nonresponders (Supplementary Fig. S3A and S3B). Similarly, the expression levels of other inhibitory checkpoints—including ICOS, Tim-3, and LAG-3—did not differ significantly between the two groups (Supplementary Fig. S3C–S3I). Notably, CD38^+^ CD8^+^ T cells, previously reported as a poor prognostic marker in HNSCC (10), were significantly elevated in nonresponders (Fig. 4A).

### Tumor antigen–reactive T cells in responders to anti–PD-1 therapy

Finally, we investigated the presence of circulating tumor antigen–reactive T cells using a short-term peptide stimulation assay. Peptides derived from EGFR and c-Met were selected to stimulate both CD4^+^ and CD8^+^ T cells (6, 7). Spontaneous IFN-γ production by PBMCs did not differ between responders and nonresponders. Similarly, peptide-induced IFN-γ responses against EGFR were comparable between the two groups (Supplementary Fig. S4). In contrast, stimulation with the c-Met–derived peptide elicited significantly higher IFN-γ production in PBMCs from responders (Fig. 4B and C). Due to limited sample availability, control peptide stimulation was performed only in a subset of patients. In these cases, stimulation with the non–tumor-related PADRE peptide induced comparable IFN-γ production between responders (CR/PR) and nonresponders (SD/PD; Supplementary Fig. S5). These findings suggest that the presence of circulating tumor antigen–reactive T cells, particularly those recognizing c-Met epitopes, may be critical for achieving favorable clinical responses to anti–PD-1 therapy.

## Discussion

In this study, we evaluated both local and systemic immune markers to identify potential biomarkers for ICIs. Neither PD-L1 expression nor stromal or intratumoral infiltration of CD4^+^ or CD8^+^ T cells predicted the response to PD-1 blockade. Although not statistically significant, the development of irAEs and the preservation of cervical lymph nodes through sparing neck dissection seemed to be associated with favorable responses. A higher proportion of peripheral lymphocytes lacking exhausted CD38^+^ CD8^+^ T cells may indicate the presence of potent antitumor lymphocytes. Furthermore, we demonstrated that tumor antigen–derived peptide–specific T-cell responses could serve as a surrogate biomarker for anti–PD-1 therapy. Because the presence of precursor effector T cells is essential for mounting a clinical response to immunotherapy, it is reasonable that preexisting tumor antigen–reactive T cells capable of being activated by PD-1 blockade represent a key determinant of therapeutic efficacy.

In the phase III trial of nivolumab, PD-L1 expression on tumor cells alone (TPS) did not serve as a biomarker capable of identifying responders (3). Because PD-L1 is also expressed on immunoregulatory cells, including myeloid-derived suppressor cells, the combined expression of PD-L1 on tumor and surrounding immune cells (CPS) has been considered a surrogate marker for ICI efficacy. On the basis of a phase III clinical trial, CPS is now used as a clinical biomarker for pembrolizumab (2). Although CPS-based stratification is useful for identifying long-term survivors who may subsequently receive second- or later-line therapies, CPS was not predictive of treatment response to PD-1 blockade, consistent with the results of the present study. Thus, the current standard biomarker—PD-L1 expression in available tumor samples—remains ineffective for reliably identifying responders to ICIs. Several explanations may account for this limitation. First, the affinity and target epitopes of clinically available anti–PD-L1 antibodies differ. In lung cancer, concordance in PD-L1 detection among three commonly used antibodies (22C3, 28-8, and SP142) has been reported to be less than 50% (11). Concordance across assays is likewise only moderate to poor for both TPS and CPS in HNSCC, suggesting that PD-L1 detection may vary substantially depending on the staining method used (12). Moreover, PD-L1 expression is readily influenced by exogenous factors—such as IFN-γ produced by infiltrating immune cells, chemotherapy, or irradiation (13)—indicating that PD-L1 status in primary tumor samples may not reflect the current PD-L1 profile of R/M HNSCC, as shown in our previous work (5). In addition, PD-L1 expression in HNSCC is highly heterogeneous, even within the same tumor (14). Although PD-L1 expression alone does not seem to represent a reliable predictive biomarker for detecting responders to ICIs, we previously demonstrated that the combined assessment of PD-L1 and PD-L2—the other ligand of PD-1—improves identification of long-term survivors treated with nivolumab (15).

We examined PD-1 expression on T cells in this study and found that PD-1 levels on both CD4^+^ and CD8^+^ T cells were not predictive of clinical responses to ICIs. Because PD-1 expression was assessed using peripheral blood samples, we also evaluated PD-1 expression in tissue specimens; however, neither peripheral nor tissue PD-1 expression correlated with the efficacy of PD-1 blockade. Similar to PD-L1, PD-1 is an unstable marker. Although PD-1 is widely recognized as a negative immune checkpoint, most activated T cells transiently express PD-1 (16). Thus, although the presence of PD-1^+^ T cells or NK cells within the tumor microenvironment at the time of PD-1 blockade is required for drug engagement, PD-1 expression in peripheral blood—or expression detected in archived samples—does not reliably reflect treatment responsiveness. We further analyzed additional negative immune checkpoints, including ICOS, Tim-3, LAG-3, and CD38, and found that peripheral CD38^+^ CD8^+^ T cells were associated with nonresponsiveness to ICIs. CD38^+^ CD8^+^ T cells are considered subprimed or dysfunctional T cells that may contribute to resistance to anti–PD-1 therapy and therefore may serve as a negative predictive biomarker (17, 18). CD38 expression may reflect either activation or dysfunctional states depending on the immunologic context, and further characterization using multiparameter analyses is required. Although multicolor flow cytometry to assess coexpression patterns of inhibitory receptors was not performed in this study due to technical limitations, simultaneous expression of several negative checkpoints—such as PD-1, LAG-3, and Tim-3—could help detecting exhausted T cells, which may hinder the expansion of competent antitumor T-cell populations (19, 20).

Lymphocytes, including cytotoxic CD8^+^ T cells, CD4^+^ T cells, and NK cells, are the major effector populations responsible for tumor elimination, whereas neutrophils play a central role in immune suppression. Accordingly, a high lymphocyte count and low neutrophil count constitute an ideal immunologic environment for achieving clinical responses with PD-1 blockade in HNSCC, as supported by previous reports (21). A drawback of using lymphocyte counts, neutrophil counts, or NLR as biomarkers is that standardized cutoff values are difficult to determine due to substantial interindividual variability. Interestingly, although absolute lymphocyte and neutrophil counts and NLR were not associated with treatment response, their respective percentages were. This discrepancy may reflect differences in how these metrics capture the systemic immune environment. Percentage-based measures may better represent the relative distribution of immune cell populations.

An advantage of assessing peptide-specific T-cell responses in peripheral blood is that the current status of antitumor immunity can be directly evaluated without requiring an additional biopsy. Because c-Met is broadly expressed in HNSCC, including circulating tumor cells (22), and because cytotoxic CD4^+^ T cells can directly kill tumor cells in addition to CD8^+^ T cells, we selected a c-Met–derived peptide that contains both CD4 and CD8 epitopes (6). Interestingly, c-Met–derived peptide responses, but not EGFR-derived peptide responses, were associated with clinical outcomes. Reanalysis of HLA binding affinity suggested that the c-Met peptide may exhibit relatively higher binding to commonly expressed HLA-A and HLA-DR alleles (Supplementary Table S1). Consistent with this, our previous studies demonstrated that c-Met–derived peptide–reactive T-cell clones were generated at a higher frequency than EGFR-derived peptide–reactive clones, suggesting greater immunogenicity (6, 7). These differences in antigenicity may contribute to the differential association observed in this study. However, these findings remain exploratory and require further validation.

Compared with neoantigen-based approaches—which require personalized identification, are costly, and are time-consuming—the use of tumor-associated antigens offers practical advantages. We found that only responders mounted detectable peptide-reactive T-cell responses, suggesting that reactivity to tumor antigen–derived peptides may serve as a promising surrogate biomarker in ICI therapy. A further advantage of this method is its broad applicability, as changing the peptide allows the approach to be adapted to different tumor types. Moreover, the short-term incubation protocol used in this study enables identification of responders within 2 weeks, which is comparatively rapid for evaluating peptide-specific T-cell responses. As IFN-γ release assays are already widely used in clinical practice for infectious diseases (23), IFN-γ detection in response to tumor-derived antigens may represent a practical and feasible method in tumor immunology as well. In a subset of patients with sufficient PBMC availability, peptide stimulation with PADRE—a non–tumor-specific universal epitope—induced IFN-γ production in all tested cases, including nonresponders. This suggests that general T-cell reactivity was preserved regardless of treatment response and that the differences observed with tumor-derived peptides may not be solely explained by overall immune competence. However, because this analysis was limited, and PADRE represents a strong non–tumor antigen, it remains unclear whether tumor-specific CD4^+^ T cells are required for predicting response or whether more broadly reactive, low-avidity T-cell responses may also serve as potential biomarkers. Further studies are needed to clarify this issue. Interestingly, neck dissection was not performed in all patients who responded to ICIs. Given that lymphocytes are primed in lymph nodes, preservation of cervical lymph nodes might be important for mounting effective antitumor T-cell responses. Consistent with this concept, the KEYNOTE-689 trial demonstrated that neoadjuvant anti–PD-1 therapy prior to surgery, including neck dissection, can induce potent antitumor T-cell responses and lead to prolonged survival (24). Further investigations are needed to determine the impact of lymph node preservation on the immune reactivity to ICIs.

In addition to direct immune-related biomarkers, several surrogate markers that reflect a patient’s systemic condition may have potential utility in identifying responders to immunotherapy. Although these factors were not associated with treatment response in the pretreatment assessment, high serum albumin and low CRP levels during the treatment could be associated with ICI responders in this study. Tada and colleagues (18) reported that elevated serum albumin is associated with favorable prognosis in patients with HNSCC treated with nivolumab. Because low albumin and high CRP are characteristic features of cachexia (25), a condition that can impair the efficacy of nivolumab (26), appropriate nutritional management or blocking IL-6 to mitigate cachexia may help enhance the therapeutic benefits of ICIs. However, it is also possible that patients who do not respond to ICIs subsequently develop cachexia as their tumors progress. Although laboratory parameters measured 1 month after treatment initiation are likely influenced by treatment response and disease progression, and therefore may reflect on-treatment immune or clinical dynamics rather than true predictive baseline biomarkers, further studies are required to clarify the causal relationship between cachexia and resistance to PD-1 blockade, as this remains a “chicken-and-egg” question.

Several limitations of this study should be acknowledged. First, the sample size was small, and this retrospective, single-institution study should be interpreted as hypothesis-generating. Given the exploratory nature and multiple comparisons, adjustments for multiplicity were not performed, and the findings should be interpreted with caution. Although T-cell responses to tumor antigen–derived peptides were observed predominantly in responders, this finding requires validation in larger, independent cohorts. In addition, the peptide-reactive T-cell assay used in this study requires freshly isolated PBMCs and immediate functional analysis, which may limit its standardization across institutions. Furthermore, there are currently limited studies investigating circulating tumor antigen–reactive T cells in HNSCC, and no established external datasets are available for validation of this specific assay. Because only a limited amount of peripheral blood was available from each participant, we were unable to determine whether the peptide-specific T-cell responses originated from CD4^+^ or CD8^+^ T cells, and HLA haplotyping was not performed in all cases. Although the peptide used in this study was designed as a promiscuous epitope capable of binding to multiple HLA-DR alleles (6), further studies incorporating multiple tumor-derived epitopes may improve coverage and generalizability. In addition, because non–tumor antigen control peptides were not included, it remains possible that the observed responses reflect general immune competence rather than strictly tumor-specific immunity. Although the presence of irAEs in responders suggests a link between immune activation and treatment efficacy, further investigation is required to determine the optimal antigen targets and assay conditions for evaluating peptide-reactive T-cell responses as biomarkers of PD-1 blockade. Finally, although anti–PD-1 monotherapy remains an important treatment option in selected patients, expanding analyses to larger cohorts and additional tumor types will be necessary to determine the broader applicability of these findings.

### Conclusions

In this study, we found that the presence of tumor antigen–reactive T cells was associated with favorable responses to anti–PD-1 therapy. The occurrence of irAEs, a high lymphocyte percentage, a low neutrophil percentage, and a low frequency of CD38^+^ CD8^+^ T cells were also identified as favorable biomarkers. Future studies incorporating appropriate control antigens and standardized assays in larger, multicenter cohorts will be necessary to validate these findings and clarify their clinical utility.

## Supplementary Material

Supplemental Table 1 Binding affinity of EGFR peptide and c-Met peptide to HLA molecules

Supplemental Figure 1 PBMCs were gated on lymphocytes, followed by doublet exclusion and identification of CD4^+^ and CD8^+^ T cells. Expression of CD38 and checkpoint molecules (PD-1, ICOS, Tim-3, LAG-3) was analyzed. Gates were defined using negative controls.

Supplemental Figure 2 Representative immunohistochemical images EGFR staining in tumor tissues from responders (CR/PR) and non-responders (SD/PD). Magnification: ×200. ns: not significant.

Supplemental Figure 3 Scatter plots showing the expression of negative checkpoints in CD4+ or CD8+ T cells evaluated by flowcytometry in responders (CR/PR; red) and non-responders (SD/PD; blue). The following markers were assessed: PD-1, ICOS, Tim3, LAG3, and CD38. Horizontal bars represent median values. Statistical differences were assessed using the Mann–Whitney U test. *P < 0.05. ns: not significant.

Supplemental Figure 4 PBMCs were stimulated with the EGFR–derived peptide, resulting in a comparable IFN-γ production in both CR/PR and SD/PD groups. ns: not significant.

Supplemental Figure 5 PBMCs were stimulated with the PADRE–derived peptide, resulting in a comparable IFN-γ production in both CR/PR and SD/PD groups. ns: not significant.

## Data Availability

All data relevant to the study are included in the article or uploaded as supplementary information.
