# Supplementary material for: Peptide-Reactive T-cell Response as a Novel Biomarker in Patients with Head and Neck Cancer Treated with Anti–PD-1 Antibody
Source: Cancer Res Commun. 2026 Jul 13;6(7):1656–64. doi: 10.1158/2767-9764.CRC-25-0796 (PMC13359030; doi:10.1158/2767-9764.CRC-25-0796)
Supplement: Supplemental Table 1 — Binding affinity of EGFR peptide and c-Met peptide to HLA molecules [file crc-25-0796_supplemental_table_1_suppst1.docx]

**Supplemental Table 1. Binding affinity of EGFR peptide and c-Met peptide to HLA molecules**

|  | | |
| --- | --- | --- |
|  | EGFR875-889 | c-Met817-831 |
|  | KVPIKWMALESILHR | TKAFFMLDGILSKYF |
| HLA-A*02:01 | 3.8 | 0.93 |
| HLA-A*24:02 | 0.71 | 4.4 |
| HLA-DRB1*01:01 | 8.2 | 0.45 |
| HLA-DRB1*03:01 | 29 | 11 |
| HLA-DRB1*04:01 | 13 | 7.8 |
| HLA-DRB1*07:01 | 22 | 29 |
| HLA-DRB1*11:01 | 23 | 9.5 |
| HLA-DRB1*15:01 | 17 | 21 |
| *Prediction method: NetMHCpan EL 4.1, Percentile Rank (Low rank = good binder). Immune Epitope Database (IEDB). Available at: https://www.iedb.org  (accessed April 3, 2026). | | |
