# Supplementary material for: Peptide-Reactive T-cell Response as a Novel Biomarker in Patients with Head and Neck Cancer Treated with Anti–PD-1 Antibody
Source: Cancer Res Commun. 2026 Jul 13;6(7):1656–64. doi: 10.1158/2767-9764.CRC-25-0796 (PMC13359030; doi:10.1158/2767-9764.CRC-25-0796)
Supplement: Supplemental Figure 1 — PBMCs were gated on lymphocytes, followed by doublet exclusion and identification of CD4+ and CD8+ T cells. Expression of CD38 and checkpoint molecules (PD-1, ICOS, Tim-3, LAG-3) was analyzed. Gates were defined using negative controls. [file crc-25-0796_supplemental_figure_1_suppsf1.pdf]

# Supplemental Figure 1. Representative flow-cytometric data.

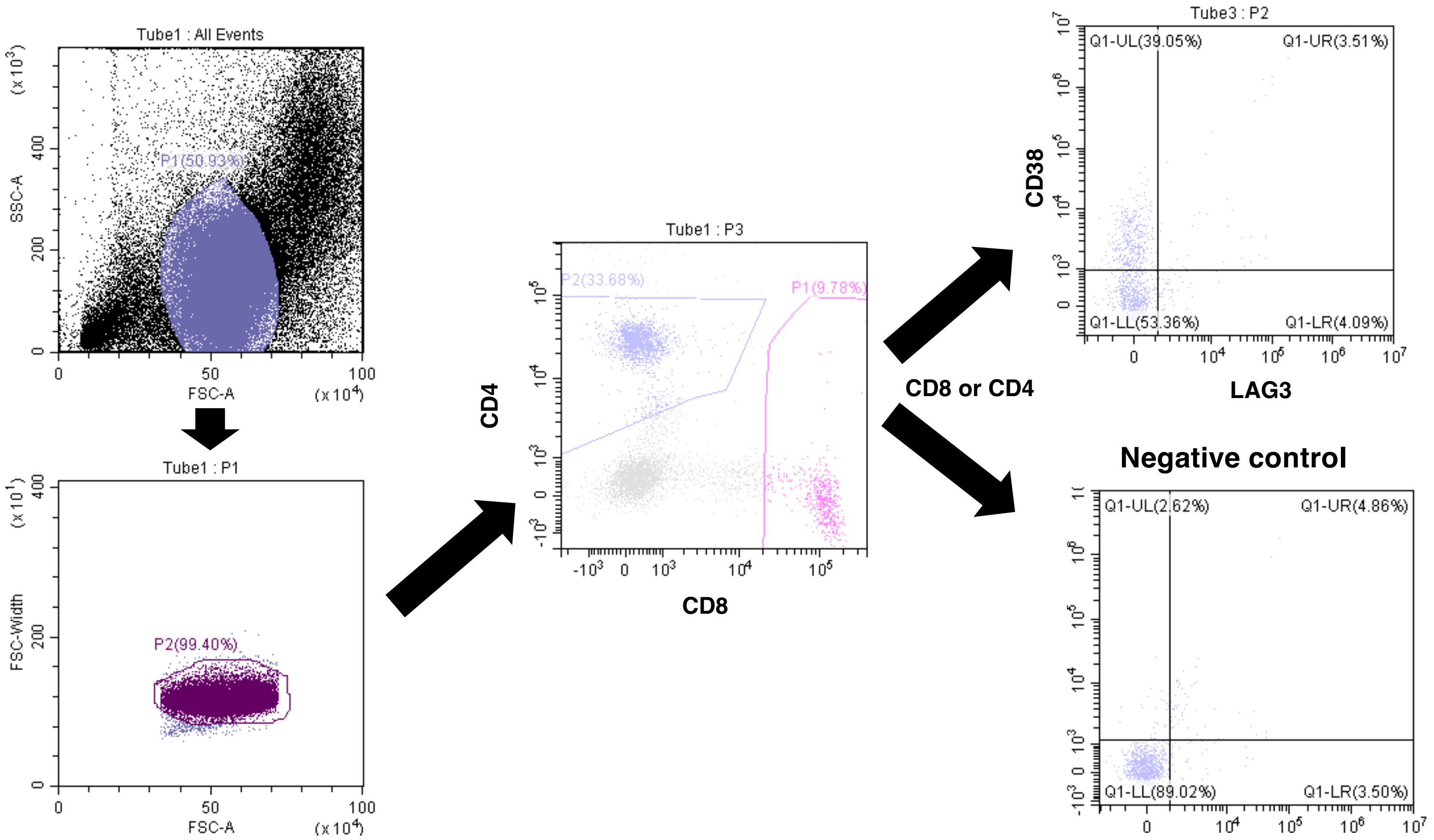


Peripheral blood mononuclear cells (PBMCs) were first gated on lymphocytes based on forward scatter (FSC-A) and side scatter (SSC-A), followed by exclusion of doublets using FSC-A versus FSC-width. $CD4^{+}$ and $CD8^{+}$ T-cell populations were subsequently identified. Expression of CD38 and other immune checkpoint molecules (PD-1, ICOS, Tim-3, and LAG-3) was analyzed within $CD4^{+}$ or $CD8^{+}$ T-cell subsets. Positivity thresholds were defined using negative control samples (unstained or isotype controls), and gates were set to exclude background fluorescence. Cells with fluorescence intensity above this threshold were defined as positive. Representative gating plots, including negative control samples, are shown.
