# Supplementary material for: Peptide-Reactive T-cell Response as a Novel Biomarker in Patients with Head and Neck Cancer Treated with Anti–PD-1 Antibody
Source: Cancer Res Commun. 2026 Jul 13;6(7):1656–64. doi: 10.1158/2767-9764.CRC-25-0796 (PMC13359030; doi:10.1158/2767-9764.CRC-25-0796)
Supplement: Supplemental Figure 2 — Representative immunohistochemical images EGFR staining in tumor tissues from responders (CR/PR) and non-responders (SD/PD). Magnification: ×200. ns: not significant. [file crc-25-0796_supplemental_figure_2_suppsf2.pdf]

Supplemental Figure 2. EGFR staining for tumor in responders (CR/PR) and non-responders (SD/PD).

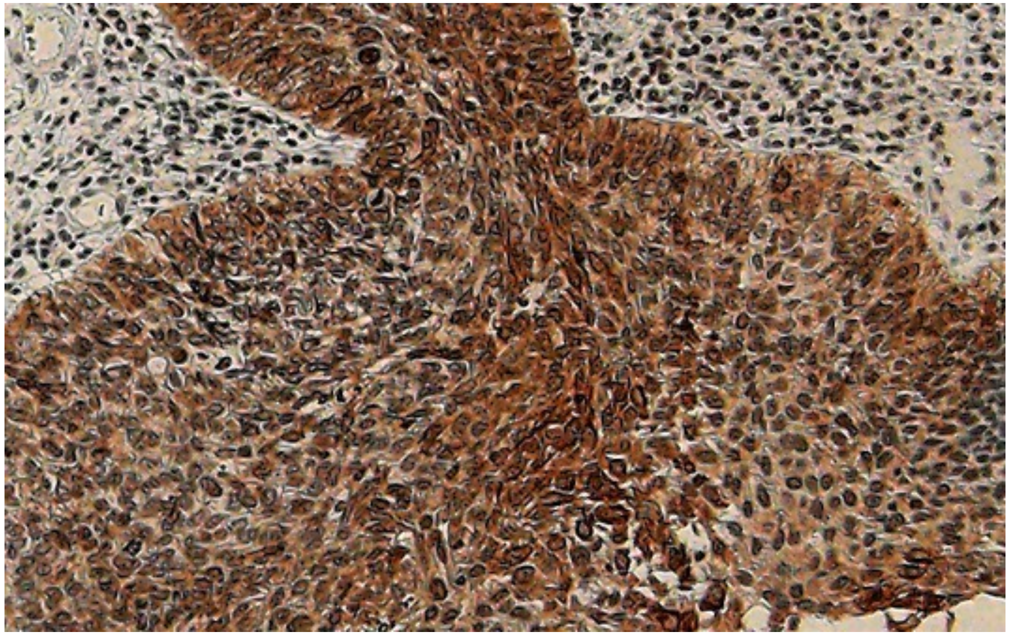

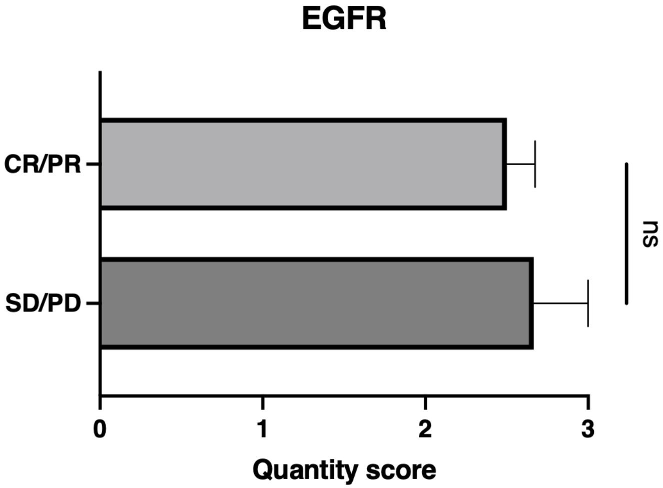

Representative immunohistochemical images EGFR staining in tumor tissues from responders (CR/PR) and non-responders (SD/PD). Magnification: ×200. ns: not significant.
