# Supplementary material for: Peptide-Reactive T-cell Response as a Novel Biomarker in Patients with Head and Neck Cancer Treated with Anti–PD-1 Antibody
Source: Cancer Res Commun. 2026 Jul 13;6(7):1656–64. doi: 10.1158/2767-9764.CRC-25-0796 (PMC13359030; doi:10.1158/2767-9764.CRC-25-0796)
Supplement: Supplemental Figure 3 — Scatter plots showing the expression of negative checkpoints in CD4+ or CD8+ T cells evaluated by flowcytometry in responders (CR/PR; red) and non-responders (SD/PD; blue). The following markers were assessed: PD-1, ICOS, Tim3, LAG3, and CD38. Horizontal bars represent median values. Statistical differences were assessed using the Mann–Whitney U test. *P < 0.05. ns: not significant. [file crc-25-0796_supplemental_figure_3_suppsf3.pdf]

Supplementary Figure 3. Comparison of peripheral lymphocyte markers between responders (CR/PR) and non-responders (SD/PD).

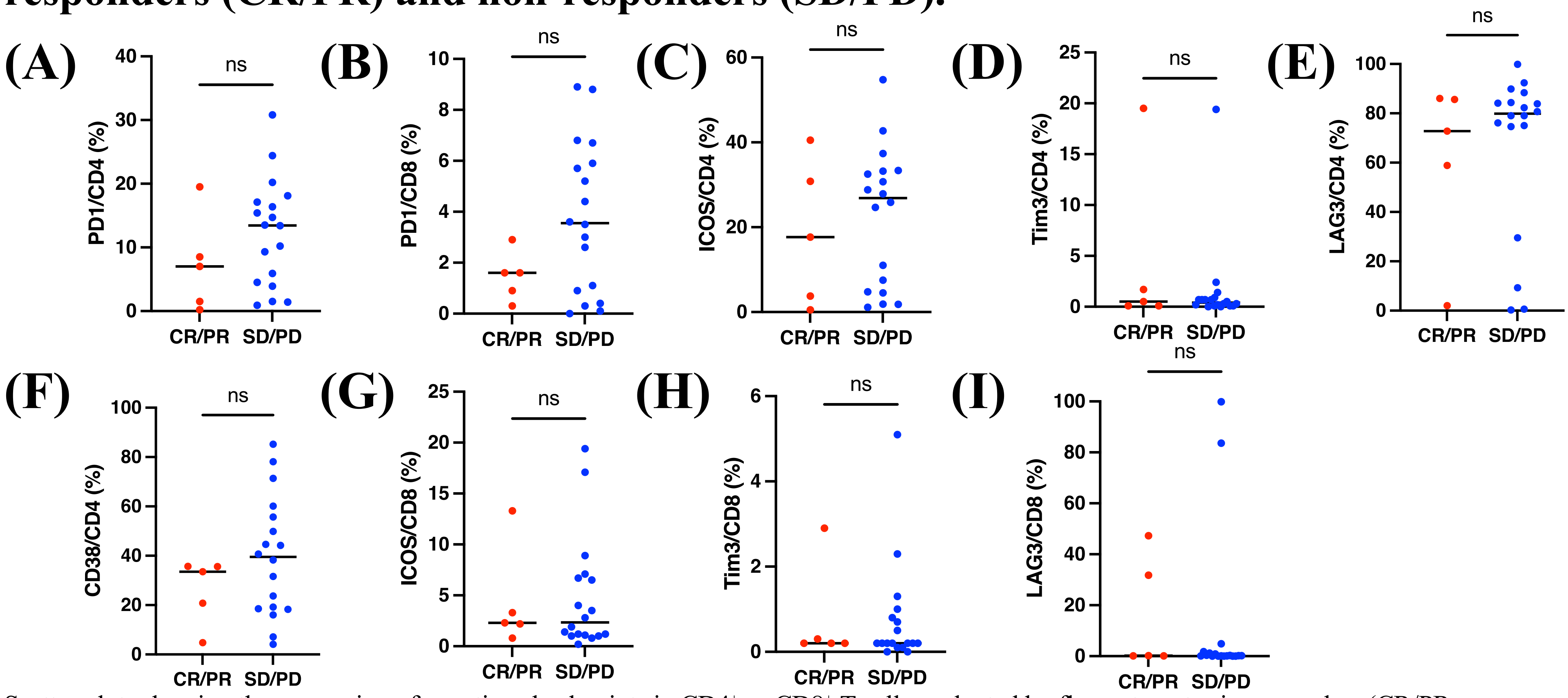

Scatter plots showing the expression of negative checkpoints in $CD4^+$ or $CD8^+$ T cells evaluated by flowcytometry in responders (CR/PR; red) and non-responders (SD/PD; blue). The following markers were assessed: PD-1, ICOS, Tim3, LAG3, and CD38. Horizontal bars represent median values. Statistical differences were assessed using the Mann–Whitney U test. *P < 0.05. ns: not significant.
