# Supplementary material for: Peptide-Reactive T-cell Response as a Novel Biomarker in Patients with Head and Neck Cancer Treated with Anti–PD-1 Antibody
Source: Cancer Res Commun. 2026 Jul 13;6(7):1656–64. doi: 10.1158/2767-9764.CRC-25-0796 (PMC13359030; doi:10.1158/2767-9764.CRC-25-0796)
Supplement: Supplemental Figure 5 — PBMCs were stimulated with the PADRE–derived peptide, resulting in a comparable IFN-γ production in both CR/PR and SD/PD groups. ns: not significant. [file crc-25-0796_supplemental_figure_5_suppsf5.pdf]

Supplemental Figure 5. IFN-γ production by PBMCs from responders and non-responders with PADRE peptide stimulation.

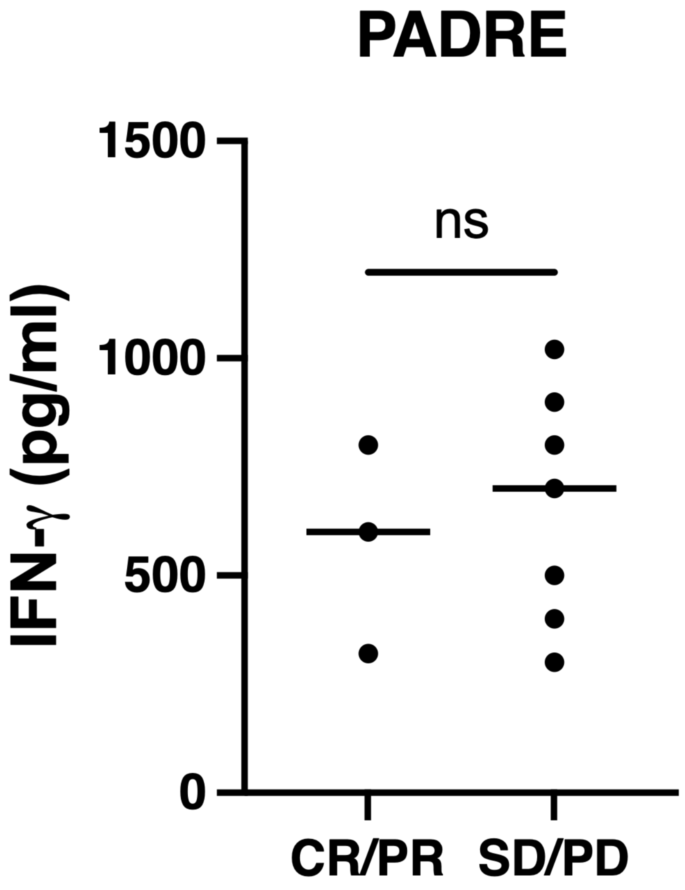

PBMCs were stimulated with the PADRE–derived peptide, resulting in a comparable IFN-γ production in both CR/PR and SD/PD groups.
ns: not significant.
